# Exploring the Impact of Emotional Eating in Children: A Narrative Review

**DOI:** 10.3390/pediatric17030066

**Published:** 2025-06-13

**Authors:** Maria Mentzelou, Sousana K. Papadopoulou, Evmorfia Psara, Olga Alexatou, Theodosis Koimtsidis, Constantinos Giaginis

**Affiliations:** 1 Department of Food Science and Nutrition, School of the Environment, University of the Aegean, 81400 Myrina, Lemnos, Greece; maria.mentzelou@hotmail.com (M.M.); fnsd21013@fns.aegean.gr (E.P.); rd.olga.alexatou@gmail.com (O.A.); cgiaginis@aegean.gr (C.G.); 2 Department of Nutritional Sciences and Dietetics, School of Health Sciences, International Hellenic University, 57400 Thessaloniki, Greece; koimtsidis1@gmail.com

**Keywords:** emotional eating, obesity, overweight, mental disorders, parental behavior, dietary habits, children, overeating

## Abstract

**Background/Objectives:** Emotional eating has been recognized as the tendency to eat in response to emotions, being recognized as a crucial risk factor for recurrent weight gain. Emotional eating has been interrelated with obesity/overweight, depression, anxiety, stress, and specific dietary habits at any stage of human life, negatively affecting human quality of life. The present study aims to summarize and explore the effects of emotional eating on children and how these effects may, in turn, influence their mental and physical health at the next stages of their lives. **Methods**: This is a narrative review of the presently existing clinical evidence concerning the impact of emotional eating in children. A comprehensive search of the most reliable online databases, e.g., PubMed, Scopus, Web of Science, and Google Scholar, was performed utilizing relevant keywords. Several inclusion and exclusion criteria were used to collect only cross-sectional, longitudinal, descriptive, and case–control surveys in children’s populations. **Results:** The current clinical evidence suggests that parental behavior may increase the risk of emotional eating in children. Several pieces of evidence also implied potential associations of emotional eating with overweight/obesity and mental disorders in children. Children’s dietary habits may also affect their risk of developing emotional eating. **Conclusions**: The prevalence of emotional eating is gradually increasing in the general population, and especially in children. Public strategies should be performed to educate parents to recognize potential emotional eating behaviors in their children and to adopt more healthy dietary habits for their children, even in the first months of their life. Educational programs should be organized in school communities to directly educate children on the benefits of healthy dietary patterns.

## 1. Introduction

Emotional eating has been recognized as the tendency to eat energy-dense and palatable foods because of adverse emotional behaviors like anxiety and depression symptomatology, negative self-concept, overeating, etc., as a means of coping with emotional fluctuations [1]. Emotional eating has also been thought of as eating in response to emotional behavior and not due to hunger, frequently as a characteristic of emotional calm or to manage adverse emotions in harmful behavior [2]. Emotional eating has also been considered a persistent behavioral phenomenon related to obesity and cardiometabolic diseases such as diabetes, hypertension, and hyperlipidemia [3,4]. Several studies have also reported that positive emotions (overeating) could affect food intake similarly to negative emotions (undereating) [5]. Emotional eating has also been related to mental diseases like depression, anxiety, stress, and eating disorders, highlighting its impact in the complex interrelationship between psychological well-being and physical health [6]. In the last few years, several studies have suggested that overeating and obesity, as well as unhealthy nutritional habits (e.g., fast food consumption), are related to emotional eating [6]. Moreover, the COVID-19 pandemic enhanced the probability of developing depressive, anxious, and stress behaviors that subsequently boosted the incidence of emotional eating [7,8]. However, most of the above evidence mainly concerns the impact of emotional eating on the general population, and especially in adults.

Several studies have explored the current incidence of emotional eating and its related factors in overweight and obese populations. A meta-analysis study including 18 studies and a total of 21,237 overweight and obese people showed that emotional eating is substantially prevalent at 44.9% [9]. Moreover, a cross-sectional study in young adults revealed that depression may be a crucial factor, which can trigger emotional eating, and emotional eating could be a critical agent which may adversely affect mindful eating [10]. Accordingly, a cross-sectional study of the NutriNet-Santé cohort, including 7378 men and 22,862 women, revealed that enhanced emotional eating was correlated with an increased intake of calorie-dense snacks like sweet and fatty foodstuffs, and these relationships were mainly higher in females diagnosed with depressive symptomatology [11]. There is also substantial evidence that individuals who were exposed to perceived stress exhibited a higher likelihood of being emotional eaters and that emotional eating could function as a regulating agent between depressive symptomatology and future body weight increase [12]. An evaluation of the prevalence of emotional eating and its relationship with anxiety and psychological distress during the COVID-19 lockdowns by a large-scale population-based study (*n* = 24,968) supported substantial evidence that psychological distress was correlated with emotional eating and a higher consumption of energy-dense foodstuffs and beverages [13]. Moreover, a case–control study showed that the emotional eating and social anxiety of obese adolescents increased compared to healthy adolescents and that the emotional eating and social anxiety of obese adolescents were interrelated to each other [14]. Emotional eating has also been related to elevated anxiety, depression, impulsivity, and sleep disorders, mainly in females, suggesting that the interrelations of psychological agents related to emotional eating could further be explored [15].

Recently, a systematic review and meta-analysis aimed to explore the relationship between depressive behavior and emotional eating in children and adolescents [16]. Thirty-seven clinical studies including 26,026 participants with a mean age of 12.4 years were subjected to a meta-analysis process. The mean effect size was found to be crucial for both cross-sectional and longitudinal surveys, indicating a direct but moderate relationship between depression symptoms and emotional eating in childhood and adolescence. Concerning prospective surveys, the relationship was higher when depression symptomatology and emotional eating were evaluated utilizing children and adolescent self-reports compared to parent reports [16]. A previous systematic review aimed to assess whether emotional eating in adolescents may be related to body weight status in this population [17]. The above meta-analysis surveys included thirteen studies, and among the six longitudinal studies, only one revealed a prospective relation of emotional eating with weight status [17]. Moreover, a narrative review revealed that the family environment, which involves parent eating behaviors and feeding practices, seems to highly affect children and adolescents’ consumption and eating behaviors [18]. In particular, parental emotional eating or emotional feeding practices may result in emotional eating in children and adolescents [18]. The currently available evidence has highlighted the significance of implementing large, longitudinal, well-organized, randomized, interventional, clinical studies to derive evidence showing advancements in eating behavior. However, most available data concerns adulthood and to a lesser extent adolescents and children. In addition, to the best of our knowledge, there is not any review article so far to summarize the currently available clinical data on the impact of the emotional eating in the children’s population. This is a strong demand, as each stage of life from childhood to adolescence and adulthood may have different characteristics concerning mental and physical health as well as the psychological status, which may affect the daily quality of life of each age-related population.

In view of the above considerations, the present study is a comprehensive narrative review, which aims to critically summarize and scrutinize the currently available clinical data regarding the impact of the emotional eating in the children’s population.

## 2. Methods

A thorough investigation of the international scientific literature was conducted on accurate scientific databases, e.g., PubMed, Scopus, Web of Science and Google Scholar, by the use of appropriate keywords like children, emotional eating, overeating, psychological disorders, eating behavior, depression, anxiety, stress, obesity, overweight, weight gain, body mass index, appetite, hunger, childhood, primary schools, etc. We comprehensively searched and retrieved any cross-sectional, longitudinal, case–control and descriptive clinical study. All studies not written in the English language, as well as animal experimental surveys were excluded. Review articles, case report surveys, commentaries and abstracts in congress/conference proceedings were also not included. The findings were classified according to their relevance and the appropriate ones were carefully selected and stated further down. Only clinical human surveys with a methodology of adequate quality were analyzed in the current review. The recovered studies were also checked for relevant surveys cited in their manuscript. The recovered surveys were thoroughly reviewed for relevant surveys reported in their manuscript.

**Figure 1:**
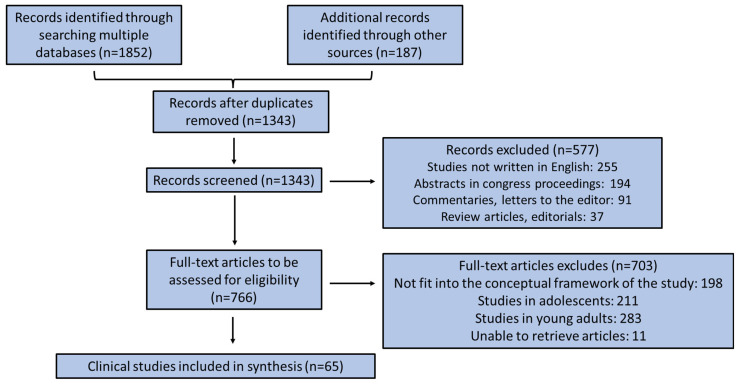
Flow chart diagram of clinical study enrollment.

All authors worked as reviewers. To increase consistency, all authors worked in pairs, and critically checked all recovered research articles, analyzed their results and adjusted the record extraction themselves prior to beginning the screening for this review. They consecutively assessed the titles, abstracts and subsequently the full manuscripts of all publications retrieved from their searches for probable related publications with a study design of adequate quality. A record monitoring form was employed mutually by two reviewers (M.M. and C.G.), who separately made a recording of the data and scrutinized the results and the charting arrangement in an iterative aspect. In Figure 1, a flow chart diagram is depicted illustrating the collection of relevant clinical surveys.

Initially, 2039 clinical studies were identified by searching multiple scientific variables. After removing duplicates, 1343 studies remained. Among them, 577 were removed based on the below exclusion criteria: (a) studies not written in English, (b) abstracts in congress proceedings, (c) commentaries and letters to the editors, and (d) review articles and editorials. Among the remaining 766 studies, 703 studies were removed based on the following exclusion criteria: (a) studies not fitting into the conceptual framework of our review study, (b) studies in adolescents, (c) studies in young adults, and (d) studies unable to be retrieved.

We finally included clinical studies assessing (a) the impact of parental behavior on children emotional eating, (b) the impact of overweight and obesity on children emotional eating, (c) the associations between mental disorders and children’s emotional eating, (d) the association of children’s dietary habits with emotional eating, and (e) the impact of other diverse factors on children’s emotional eating.

## 3. Results

### 3.1. Parental Behavior Effect on Children’s Emotional Eating

There is substantial evidence that children are more likely to adopt emotional eating when their parents consume specific foodstuffs to control their emotions in response to diverse negative circumstances such as stress. In this respect, we identified 65 relevant clinical studies, which are included in Table 1 [19,20,21,22,23,24,25,26,27,28,29,30,31,32,33,34,35,36,37,38,39,40,41,42]. In this aspect, one of the first cross-sectional studies designed to evaluate parental characteristics related to overweight and eating behaviors in preschool children [19]. In fact, 75 children aged 4–6 years and their matched parents were enrolled from community daycare centers. Parents’ BMI, nutritional restraint, and nutritional disinhibition were collected. A behavior indicator of disinhibited eating in children was applied for measuring children’s consumption when freely admitting to the consumption of palatable snack foodstuffs in the absence of appetite. Maternal dietary disinhibition and BMI positively predicted daughters’ overweight. Moreover, when maternal disinhibition and BMI were applied for predicting their children’s overweight, maternal disinhibition was found to affect the relationship of maternal BMI with children’s overweight. In addition, maternal disinhibition exhibited independent prediction when mothers’ disinhibition and their children’s free admission of consumption were utilized to predict children’s overweight. The above results supported evidence that familial impacts on children’s overweight may be different based on parental and child gender. Also, the above findings suggested that maternal nutritional disinhibition may mediate familial correspondences to the level of overweight for mothers and their children [19].

Schuetzmann et al. performed a cross-sectional study to repeat the findings from clinical samples for which families of overweight children showed harmful attributes of the parent–child connection [20]. A public-based sample of 373 fourth-grade students aged 8–11 years in Germany obtained self-reported questionnaires on the perceived parent–child relation and on eating behavior. No significant relations of children’s body weight with the parent–child relationship were noted. Unusual eating behavior was highly related to an adverse parent–child relationship regardless of children’s body weight. Thus, it was supported that the above evidence from clinical samples of overweight children cannot normally be generalized to the general overweight child population and that aberrant eating performance, and not overweight itself, may be associated with an adverse parent–child relationship in preadolescent children [20].

Another cross-sectional study aimed to evaluate the potential associations of parenting style, parental response to harmful child emotion, and family emotional expressiveness and reinforcing child emotional eating [21]. In fact, mothers (*n* = 450) completed questionnaires and their matched 6–8year-old children (*n* = 450) from the USA were interviewed. The emotional eating subscale of the Dutch Eating Behavior Questionnaire (DEBQ) was used to assess Child Emotional Eating (CEE). This study found that emotional eating was adversely predicted by reliable parenting style and family open expression of affection and emotion and directly predicted by parents reduced response to children’s harmful emotion. The above findings suggested the necessity for immediate prevention/intervention efforts managed by these parenting and family factors [21].

Only a few clinical studies have a prospective design to examine eating behaviors in childhood. Most of these studies supported evidence that eating attitudes may reasonably be constant from early childhood forwards; however, understanding individual patterns within childhood still remains scarce. Another prospective study determined which eating patterns were more likely to be recognized as difficult by parents, and their effect and relation to childhood psychopathology (emotional, behavioral, and pervasive developmental disorders) in a wide-ranging child cohort [22]. This study derived data as part of the 5- to 7-year-old follow-up of a randomly resulting subsample of the Copenhagen Child Cohort 2000. Of the available 2912, 1327 (45.6%) children aged 5–7 years and their matched parents joined this study. Parents were questioned utilizing a combined instrument evaluating eating performances and their effect. Five eating patterns were recognized (good eating/overeating, picky eating, slow/poor eating, paused eating performances, and snacking actions) among these. Picky eating and slow/poor eating were considered as a problem by more than half of parents and they also exhibited an elevated effect. Picky eating was associated with psychopathology across disorders. Emotional undereating was associated with emotional and functional somatic symptoms. A quarter of parents reported at least one eating attitude as a problem. Eating performances in a usual population cohort were diversely related to psychopathology. Picky eating was identified amongst other attitudes as exhibiting harmful correlations [22].

A more recent cross-sectional survey explored potential associations of children emotional eating with general and specific parental constructs and maternal depressive symptomatology with binge eating in a treatment-seeking sample of overweight children [23]. This survey enrolled 106 mothers and their matched children aged 8–12 years who joined a standard evaluation for assignment in a behavior interventional analysis for overeating. Mothers fulfilled self-reported measurements of their children’s emotional eating patterns, their own feeding approaches, and depressive symptomatology and binge eating. Children fulfilled self-reported measurements of their mothers’ general parental pattern. The Child Eating Behavior Questionnaire (CEBQ) was used to assess child emotional eating. This study showed that the parental variable was more intensely correlated with child emotional eating, controlling for children age and sex. Emotional feeding behavior (i.e., a tendency to give foods to soothe children’s negative emotions) was the most significant parent factor related to children’s emotional eating. These results suggested that emotional feeding attempts in parents could be collated with emotional eating in childhood. Thus, it was proposed that treatment of overweight children engaging in emotional eating may be better by targeting parental feeding methods [23].

Philips et al. performed a cross-sectional study to explore potential associations between parental behavior and children’s health-associated behavior containing physical activity, sedentary and nutritional behavior, and sleep quality. More to the point, 288 parents and their matched children (6–12 years) were finally enrolled [24]. Children completed the DEBQ. A borderline positive relationship was noted between sweet food intake incidence and “coercive control”. Moreover, a marginal negative association of fruit and vegetable intake incidence and “overprotection” was also noted. Children more commonly consumed soft drinks when their parents had lower “structure” and greater “overprotection”; for the light soft drinks distinctly, a weak positive association with “behavioral control” was noted. A borderline negative association was also reported between “emotional eating” and “structure” as well as “behavioral control” [24].

Another cross-sectional study was designed to evaluate a model in which mothers’ negative affect could be associated with mothers’ emotional eating, which in turn may be correlated with children’s emotional eating via mothers’ feeding strategies (emotional and instrumental feeding) [25]. For this purpose, 306 mothers and their matched 2-year-old children from Australia were enrolled. The mothers participated in a study evaluating symptoms of depression, anxiety and stress, mothers’ emotional eating, mothers’ feeding practices, and children’s emotional eating. The Emotional Eating subscale of the DEBQ assessed maternal emotional eating as well as emotional eating in children. Mothers’ symptomatology of depression, anxiety, and stress were associated with mothers’ emotional eating and children’s emotional eating. The first model did not have an adequate fit for the data. Modification indices showed that the model could be better when a direct path was included between mothers’ and children’s emotional eating. The resulting model proved a good fit to the data, explaining 29% of the variance in children’s emotional eating. Hence, elevated levels of negative affect and associated emotional eating in mothers could help the usage of instrumental and emotional feeding methods. The above evidence supported that mothers’ negative affect may have an indirect impact on children’s emotional eating, mainly via mothers’ own emotional eating and feeding their children to control the children’s emotions [25].

Tan et al. carried out a cross-sectional study to evaluate whether feeding for emotion regulation can mediate the association between parents’ and children’s emotional eating, and whether this association may be moderated by children’s self-regulation in eating. Ninety-five parents and their matched children aged 4.5–9 years from the USA participated in this study [26]. The parent version of the DEBQ was used to measure children’s emotional eating. To determine parents’ own negative emotional eating, the DEBQ was used. The enrolled parents responded regarding their own and their children’s emotional eating, their children’s self-regulation in eating, as well as their feeding strategies. This study found that feeding for emotion modulation was implicated in the relationship of parents’ and children’s emotional eating when children’s self-regulation in eating was decreased, but not when self-regulation in eating was increased. The above evidence demonstrated the complexity of the relationship between parental and children’s emotional eating, signifying practitioners to take into consideration both feeding practices and children’s self-regulation in eating when designing interventional programs [26].

A more recent longitudinal study assessed whether emotional eating could be induced in 5–7-year-old children, assessing whether parents’ use of excessively monitoring feeding strategies at 3–5 years of age may predict a higher consequent tendency for children to eat under conditions of mild stress at the age of 5–7 years [27]. Forty-one parents and their matched children were enrolled to take part in this prospective survey in the UK, which included parents and children experiencing consumption of a typical lunch, fulfilling questionnaire measurements of parents’ feeding methods, taking part in a research process to promote children’s emotion (or a typical process), and evaluating children’s intake of snack foodstuffs [27]. Children aged 5–7 years subjected to a moderate emotional stressor consumed considerably more calories from snack foodstuffs in the absence of hunger compared to those of the control group. Parents reporting the usage of additional foods as a reward and limitation of foods for health purposes with their children at the age of 3–5 years were more likely to have children eating more under circumstances of negative emotion at the ages of 5–7 years. Thus, parents who excessively monitor children’s foods consumption could accidentally educate their children to have confidence in palatable foods to cope with negative emotions. However, further research is recommended to determine the effects of the above evidence for children’s foods’ consumption and weight beyond the laboratory practice [27]. Moreover, no validated emotional eating questionnaires were applied [27].

A cross-sectional study aimed to examine whether attachment anxiety may be crucial maternal features which could predict parents’ reports of children’s emotional over-eating through its impacts on mothers’ disinhibited eating and emotional feeding [28]. In this respect, mothers of preadolescent children (*n* = 116) aged 3–12 years from the UK completed an internet questionnaire. Mothers’ attachment anxiety and dietary disinhibition were evaluated by the Experiences in Close Relationships questionnaire (ECRQ) and the Three Factor Eating Questionnaire (TFEQ), respectively. The Parental Feeding Strategies Questionnaire (PFSQ) and the CEBQ were utilized to measure emotional feeding and children emotional over-eating, respectively. A considerable direct effect of mothers’ attachment anxiety on children’s emotional overeating (i.e., after adjustment for mothers’ disinhibited eating and emotional feeding) was noted. A substantial indirect impact of mothers’ attachment anxiety on children’s emotional overeating via emotional feeding approaches was noted. In a subsequent model to examine bi-directional associations, the direct impact of mothers’ attachment anxiety on emotional feeding methods was not significant after adjustment for children’s emotional over-eating. Nevertheless, there was a considerable indirect impact of mothers’ attachment anxiety on emotional feeding methods through children’s emotional overeating. The above evidence emphasized the impact of mothers’ attachment anxiety on parents’ answers of abnormal eating behavior in children. The above findings could partially be ascribed to the usage of emotion feeding strategies; there could be stronger evidence for a “child-responsive” model whereby anxiously attached mothers may use these feeding practices in response to perceived emotional over-eating in the child [28].

Another cross-sectional study explored the impacts of mentalization on mothers’ and children’s obesity in Germany [29]. Children’s emotional eating was determined by the Emotional Overeating subscale from the CEBQ and mothers’ emotional eating was assessed with a subscale from the DEBQ. This study enrolled obese (*n* = 30) and normal-weight (*n* = 30) mothers and their matched children at the age of 18 to 55 months. It was shown that obese mothers’ mentalization (Reflective Functioning Scale) was comparable to that of normal-weight mothers. Moreover, mentalization exhibited no direct impact on children’s weight. Nevertheless, it was noted to have an indirect influence of mentalization through emotional eating on mothers’ but not on children’s weight and through mother–child attachment (Attachment Questionnaire-Set) on children’s weight [29].

Houldcroft et al. performed a longitudinal study evaluating the permanency and stability of preadolescent observations of their parental monitoring feeding methods (pressure to eat and restraint) over a one-year interval in the UK [30]. This study was also designed to evaluate whether experiences of parents’ feeding strategies can moderate the association of preadolescents’ eating behaviors through a prospective design. Two hundred and twenty-nine preadolescents (mean age at enrollment: 8.73 years) completed questionnaires determining their eating patterns and their awareness of parents’ feeding strategies at two time points, 12 months apart (T1 and T2). Preadolescents’ experiences of their parents’ feeding strategies stayed constant. Experiences of limitation and pressure to eat were permanent. Experiences of parents’ pressure to eat and restraint substantially diminished the associations between eating patterns at T1 and T2. The above evidence suggested that in a preadolescent population, experiences of parents’ pressure to eat and restraint of foods could further promote the development of problematic eating behaviors [30].

The parent feeding practice of using food as a reward has been directly correlated with children’s emotional overeating; however, the potential behavioral mechanisms linking these behaviors remain unknown. In this aspect, another cross-sectional study examined the mediating impact of children’s self-regulation of eating in the association between parents’ use of food as a reward and children’s emotional overeating [31]. Parents of 254 preschool children with an average age of 4.17 years completed online questionnaires concerning parental feeding methods, children eating patterns, and children’s self-regulation in eating. This study showed that the association of parents’ use of food as a reward and children’s emotional overeating was partly mediated by children’s self-regulation in eating, even after adjustment for parents’ and children’s sex, family income, and race/ethnicity. In view of the above findings, parents’ use of food as a reward may result in children’s reduced capability to control consumption, which in turn may result in elevated emotional overeating. The above evidence may have potential effects for both the prevention of disturbed eating behaviors and childhood obesity prevention programs, reinforcing the necessity to help children in learning how to self-regulate in the presence of food [31].

Munch et al. performed a cross-sectional study to investigate whether parental emotion (criticism and emotional overinvolvement) could be associated with children’s emotional eating and if this association may be mediated by children’s negative determination [32]. One hundred children at the age of 8 to 13 years in Switzerland, either healthy or experiencing binge-eating disorder and/or attention-deficit/hyperactivity disorder, completed the questionnaires, along with their parents. Children completed the emotional eating subscale of the DEBQ for 7- to 13-year-old children. Parents’ critique and, to a lower degree, parents’ emotional overinvolvement were both positively associated with children’s emotional eating, and this association was mediated by children’s negative pressure. Additional investigative analyses revealed that the mediating role of children’s negative urgency in the relationship between parental criticism and children’s emotional eating was pronounced in the clinical group of children with binge-eating disorder and attention-deficit/hyperactivity disorder, but it was almost absent in the healthy control group [32].

In a typical communal sample of 801 Norwegian children at the age of 4 years followed up at the ages of 6, 8, and 10 years, the reciprocal relationship among parental emotional feeding and child emotional eating was examined [33]. Emotional eating was evaluated utilizing the emotional overeating subscale of the Norwegian version of CEBQ. Parents’ emotional feeding was assessed using the PFSQ. This study supported evidence that elevated emotional feeding may predict increased emotional eating and vice versa after adjustment for BMI and initial levels of feeding and eating. Elevated temperamental negative affectivity (at the age of 4 years) enhanced the probability of developing emotional eating and feeding in the future [33].

A prospective study was designed to explore eating patterns of children aged 4 to 10 years in a population-based sample with the aim of recognizing parental and early life predictors of these patterns [34]. In this study, 3514 children were enrolled from The Generation R Study with frequent measurements of the CEBQ at ages 4 and 10 years in Netherlands. Patterns of emotional overeating, food responsiveness, enjoyment of food and satiety responsiveness were investigated, aiming at categorizing sub-groups of children with distinct eating behavior patterns. Parents’ and early life predictors of eating behavior patterns were also explored. This study recognized three patterns of emotional overeating (stable low (*n* = 2240); moderately elevating (*n* = 1028); strongly elevating (*n* = 246)) and five patterns of food responsiveness (stable low (*n* = 2343); high reducing (*n* = 238); moderately reducing (*n* = 679); strongly reducing (*n* = 141); stable high (*n* = 113)) from an age range of 4 to 10 years. For food enjoyment and satiation responsiveness, a similar pattern was recognized for all children. Obesogenic eating behavior patterns were associated with an elevated childbirth body weight and BMI, emotion and behavior troubles, mothers’ overweight/obesity and controlling feeding methods. The above knowledge may contribute to recognizing children at risk of developing obesogenic eating behaviors [34].

Higher eating or lower eating due to negative emotions, termed as emotional over- and undereating, occurs conventionally in children; however, research on the etiology of these eating patterns still remains scarce [35]. A large, representative public sample of Norwegian children followed up on a biennial basis from 6 to 10 years of age (*n* = 802), and child and contextual predictors (i.e., children temperament, depressive symptomatology, severe life episodes, family functioning, parents’ sensitivity and structuring) of alteration in emotional over- and undereating were investigated. The emotional overeating scale and the emotional undereating scale of the parents’ reported CEBQ were utilized to evaluate children’s emotional over- and undereating at the ages of 6, 8 and 10 years. This study revealed that reduced (temperamental) soothability and lower parent structuring at the age of 6 years could predict elevated emotional overeating at the age of 10 years and that decreased family functioning at the age of 6 years could predict more emotional undereating throughout the same interval [35].

A case–control study was conducted in 440 children aged 3–6 years in India who were separated to two groups: Group A-children with Early childhood caries (ECC) and Group B-children without ECC [36]. Dental caries was evaluated utilizing the DMFT index (Decayed, Missing and Filled Teeth). The parents of children in both groups fulfilled the CEBQ and PFSQ. It was observed that there was a positive relationship between food avoidance subscales of CEBQ (Satiety Responsiveness, Food Fussiness, Slowness in Eating, and Emotional Undereating) and several food-approaching subscales (Desire to Drink and Emotional Overeating) and dental caries status. It was additionally found that parents’ feeding patterns, like encouragement and instrumental feeding, caused a reduction in dental care of children when compared to control and emotional feeding. Thus, it was assumed that certain eating and feeding behaviors may appear to be related to the development of ECC, and such behaviors can be effectively recognized by CEBQ and PFSQ [36].

Both genetic and environmental factors can enhance complicated multidimensional relationships of mothers’ and children’s eating behaviors, mothers’ feeding strategies and the probability of childhood obesity. In this aspect, Miller et al. explored potential cross-sectional association among mothers’ and children’s eating behaviors, and examined if mothers’ feeding methods may be implicated in these associations [37]. This survey was conducted on 478 Australian mothers of 5–10-year-old children. Mothers’ emotional overeating and food responsiveness were directly related to the corresponding childhood eating behavior. Both the associations between mothers’ and children’s emotional overeating and between mothers’ and children’s food responsiveness were partly implicated, utilizing foods as a reward and overt constraint. The above findings suggested that feeding practices may exert an impact on describing the agreement of mothers’ and children’s eating behaviors. Additionally, this study highlighted the necessity for applying interventional activities that may reinforce parents to recognize the above eating behaviors in themselves and their children and recognize in what way these could possibly affect the feeding strategies they can apply [37].

The purpose of another cross-sectional study was to investigate potential relationships among parents’ stress, feeding methods, and awareness of children’s eating behaviors throughout the COVID-19 confinement [38]. The CEBQ was used to assess parents’ perceptions of children’s eating behaviors. Parents (*n* = 284) of children at the age of 4 to 6 years completed a cross-sectional online study throughout the beginning of pandemic-associated stay-at-home directives in the USA. Elevated parenting stress was related to less desirable feeding practices, involving greater likelihood of high consumption of foodstuffs for emotional modulation, foods as a reward, and pressure to eat, and reduced encouragement of a balanced diet. Elevated parenting stress was also related to enhanced experiences that children presented problematic eating behaviors, involving elevated probability of increased high food fussiness and reduced enjoyment of food. Parents reporting that their parenting stress increased, and elevated parents’ stress was related to additional common pressure to eat and less frequent control of their children’s nutrition. Hence, parenting stress during the pandemic was related to the consumption of foods for emotion and behavior modulation and awareness that children presented problematical eating behavior [38].

Stone et al. performed a cross-sectional study to explore whether the relationship between parent and childhood emotional eating may be affected by parenting feeding practices, and whether the degree of this association may vary as a pattern of child temperament [39]. Two-thousand forty-four mothers of children at the age of 3 to 5 years completed questionnaires concerning their emotional eating, feeding methods, their child’s emotional eating and temperament. The DEBQ was applied for measuring the parental emotion eating subscale and the CEBQ was used to evaluate child emotional overeating. This study indicated that parenting usage of foods to control child emotions totally intervened in the association between parents’ and children’s emotional eating. Moreover, utilizing foodstuffs as a reward and restricting foods for health purposes were partly implicated in the above association. This survey also indicated that the mediated association between parents’ and children’s emotional eating by the usage of foods as a reward and constraint of foods for health purposes varied as an action of children’s negative affect, in which enhanced children’s negative affect reduced the above interventions. The above results suggested that children’s emotional eating could be ascribed to the interrelations among elevated parents’ emotional eating, the usage of foods as a reward, constraint of foods for health purposes and negative affective temperaments. However, higher usage of foods for emotion management seems to predict higher child emotional eating independently of children’s temperament [39].

Moreover, Stone et al. performed another cross-sectional study to evaluate whether the previously identified mediating relationship between maternal emotional eating and child emotional eating through maternal use of food as a reward, food for emotion regulation, or restriction of food for health reasons may vary as a function of child food approach [40]. One hundred eighty-five mothers of children aged between 3 and 5 years were enrolled from social media advertisements in the UK. The DEBQ was used to assess the parental emotional eating subscale and the CEBQ was applied for measuring child emotional overeating. This survey indicated that the association of mothers’ emotional eating with children’s emotional eating was facilitated by mothers’ utilization of foods as a reward, but just for children with elevated food approach tendencies. The above survey also showed that the association of mothers’ emotional eating and children’s emotional eating was facilitated by mothers’ use of constraint for health purposes, but only when children were characterized by intermediate to high food approach tendencies. The intergenerational transmission of emotional eating via the usage of foods as a reward and food constraint could be impaired when child had elevated food approach behaviors [40].

Furthermore, a laboratory-based experimental survey investigated if manipulated maternal mood may impact consequent eating and parents’ feeding in overweight or obese mothers having children at the age of 3 to 5 years in a laboratory-related experimental setting [41]. Overweight and obese mothers and their children randomly participated in either acute distress or control groups. This survey did not find any difference between the two groups concerning calories served or consumed. Mothers within both groups reporting elevated emotional eating served themselves and their children a smaller amount of food, and mothers also ate a lesser amount of food. Thus, this study suggested that mothers being characterized by emotional eating trends could feed their children fewer foods throughout the intervals of acute distress [41].

A cross-sectional study examined the potential relationship between mothers’ immigration status and emotional eating in children aged 10–11 years from Taiwan, exploring the mediating impact of health literacy and feeding practices [42]. Emotional eating was measured utilizing a subscale of the Three-Factor Eating Questionnaire-Revised (TFEQ) [42]. Children of mothers with foreign nationalities showed elevated emotional eating scores compared with those with native-born mothers. These mothers also exhibited considerably reduced health literacy levels. Additionally, they were more persuaded to utilize rewarding and pressure-to-eat feeding methods and showed decreased trends toward monitoring and restriction. This survey also supported evidence that maternal foreign nationality affected children’s emotional eating mainly through enhancing rewarding and pressure-to-eat methods combined with decreased health literacy that eventually lowered monitoring strategies [42].

### 3.2. Association of Overweight and Obesity with Children’s Emotional Eating

It is currently well-recognized that emotional eating may predispose an individual to obesity. In this aspect, there are several clinical studies evaluating the impact of overweight, obesity and BMI in children’s emotional eating, which are included in Table 2 [43,44,45,46,47,48,49,50,51,52,53,54,55,56,57,58,59,60]. In this aspect, Braet et al. performed a cross-sectional study to evaluate whether there are variations in eating behavior concerning obese and non-obese children [43]. Applying the parents’ version of the DEBQ, this study revealed an approving response to this issue. The Perceived Competence Scales for Children (PCSC) was applied for evaluating emotional eating of children. The scores for 292 obese children aged 9–11 years from Belgium were considerably elevated concerning the levels for emotional, external and restrained eating behavior. This survey found significant associations between emotional eating and negative feelings of physical competence; between external eating and negative feelings of self-worth; and between both eating styles and diverse dimensions of problematic behaviors. No association was noted between external eating and control status. Elevated levels on both scales were associated with greater caloric intake. These findings supported evidence that DEBQ could be used as a screening instrument for assessing eating styles of obese children [43].

A multicenter cross-sectional survey of risk factors for obesity developed an instrument for determining emotion-induced eating in children and evaluated assumptions concerning the relationship between emotion-induced eating and food intake as well as adiposity in preadolescent children [44]. In fact, the enrolled USA children were 1213 black girls and 1166 white girls aged from 9 to 10 years at survey admission. Black girls had considerably elevated emotion-induced eating scores compared to white girls. For white girls, but not for black girls, emotion-induced eating was related to elevated consumption of sucrose. In both races, a moderate negative relation was noted between BMI and emotion-induced eating [44].

The purpose of another survey was to evaluate the relationship of CEBQ scores with BMI in Portuguese children [45]. Applying a cross-sectional design, 240 children at the age of 3–13 years were enrolled from clinical and community-related settings. Parents completed the CEBQ to indicate their children’s eating style for three ‘food approach’ and four ‘food avoidant’ subscales. Hierarchical regression analyses adjusting for sex, age and socioeconomical level revealed that all CEBQ subscales were substantially associated with BMI z-scores. Food approach scales were also positively associated with BMI z-scores and food avoidance negatively related. The above findings supported evidence for the utility of CEBQ to additionally explore eating approach as a behavioral path to obesity [45].

Another cross-sectional study was performed in 135 parents and their matched children aged 6 and 7 years from the Netherlands. This survey aimed to evaluate potential relationships of the mean scale levels of the CEBQ with children’s BMI [46]. Children’s BMI was transformed into standardized z-scores, adjusted for children’s sex and age to evaluate the relationship of mean scale levels with children body weight status. BMI z-scores were positively associated with the ‘foods’ approach’ subscales of the CEBQ (food responsiveness, enjoyment of foods, emotional overeating) and negatively with ‘foods’ avoidant’ subscales (satiety responsiveness, slowness in eating, emotional undereating, and food fussiness). Substantial associations with children’s BMI z-scores were noted for food responsiveness, enjoyment of foods, satiety responsiveness and slowness in eating [46].

Webber et al. also performed a study with a cross-sectional design in a community setting to assess whether quantitative variation in eating behavior patterns may show a rated relationship with body weight in children [47]. Relevant data were retrieved from the Physical Exercise and Appetite in CHildren Study (PEACHES) or the Twins Early Development Study (TEDS) concerning 406 families. Children aged 7 to 9 years (PEACHES) and 9 to 12 years old (TEDS) were enrolled. Eating behavior traits were assessed with CEBQ, completed by the parents on behalf of their children. Satiety Responsiveness/Slowness in Eating and Food Fussiness indicated a gradual negative relation to body weight. Food Responsiveness, Enjoyment of Foods, Emotional Overeating and Desire to Drink were interconnected. All effects were retained after adjusting for age, gender, ethnicity, and parents’ education. Body weight was not related to Emotional Undereating. The above findings supported the perception that approach-associated and avoidance-related appetitive behaviors may be inversely associated with adiposity, and not completely related to obesity. This study provided the hypothesis that early assessment of the above behaviors may be considered as factors related to the vulnerability to body weight increase [47].

In a longitudinal birth UK cohort, maternal ratings of children’s appetite made at 6 weeks, 12 months and 5–6 years were correlated with one another and with subscales from the CEBQ at 5–6 years and BMI at 6–8 years [48]. From the original birth cohort of 1029 children, responses to the appetite question were available for 811 at 6 weeks and 620 at one year. CEBQ questionnaires were completed by mothers for 506 children aged 5 to 6 years and answers to the appetite question were obtained for 492 children. Substantial relationships were noted between the children’s appetite scores. Appetite scores in childhood were associated with the CEBQ subscale levels at the age of 5–6 years to a low level, but not with the BMI at the age of 6–8 years. The appetite score at 5–6 years and three of the CEBQ subscales were independently related to BMI. Children presenting elevated scores of Emotional Over-Eating and Desire to Drink presented increased BMIs, and children presenting elevated scores of Satiety Responsiveness exhibited reduced BMIs. The above findings provided additional indications that there may be parallel relationships of appetite scores in childhood with BMI, suggesting that appetite scores in childhood could be associated merely at a weak incidence to future appetite measurements and cannot predict future BMI [48].

Haycraft et al. performed a cross-sectional study to examine the relationships of eating behaviors with temperament in young children. Mothers (*n* = 241) of children at the age of 3 to 8 years from the UK obtained measurements of their children’s eating behaviors as well as their temperament, collecting data concerning their children’s height and weight [49]. CEBQ was used to examine children’s food approach and food-avoidant eating behaviors. Children presenting elevated emotional temperaments showed higher food-avoidant eating patterns. Shyness, sociability and activity were not related to children’s eating behaviors. Elevated child BMI was associated with higher food approach eating behaviors; however, BMI was not related to children’s temperament. Notably, the children in this sample commonly exhibited healthy weights and the fact that the relationships of temperament with eating behaviors may be higher may be ascribed to being over- or underweight. The authors suggested that additional research needs to closely investigate whether emotional temperaments may affect children eating behavior [49].

The purpose of another cross-sectional survey was to investigate if various individual eating behaviors were different among body weight status groups in 1730 children aged 4–5 years from Canada [50]. Parents completed the CEBQ. Significant differences were noted between body weight status groups for food responsiveness, emotional overeating, enjoyment of foods, satiety responsiveness, slowness in eating, and food fussiness. No substantial differentiations were noted regarding the desire to drink or emotional undereating. An inspection of mean scores indicated positive linear patterns grouped by body weight for food responsiveness and enjoyment of foods and arranged negative linear patterns by body weight for satiety responsiveness, slowness in eating, and food fussiness [50].

Body weight troubles that appear in the initial stages of life tend to persist. Behavioral research during this interval can offer useful data on the modifiable etiology of no healthy weight. In this respect, Jansen et al. replicated evidence from previous small-scale surveys by evaluating if different features of preschool children’s eating behavior and parents’ feeding strategies could be related to BMI and weight status, including underweight, overweight and obesity [51]. In fact, cross-sectional data on the CEBQ, Child Feeding Questionnaire (CFQ) and BMI was available for 4987 children aged 4 years who participated in a population-based cohort in the Netherlands. Greater levels of children’s Foods Responsiveness, Enjoyment of Foods and parental Restriction were related to a greater BMI independently of several confounding factors. Emotional Undereating, Satiety Responsiveness and Fussiness of children as well as parental Pressure to Eat were inversely associated with children’s BMI. Analogous trends were noted with BMI. A part of the relationship of children’s eating behaviors with BMI was accounted for by parental feeding practices, while children’s eating behaviors in turn explained part of the relation between parental feeding and child BMI. This study provides substantial evidence of how young children’s eating behaviors and parents’ feeding behaviors could be different between children with normal weight, underweight and overweight. The increased incidence of under- and overweight amongst preschoolers highlighted the need to perform prevention interventions targeting unhealthy weights at the earlier stages of child life. Even if prospective surveys are crucial to establish causal effects, attempts for prevention or treatment of no healthy child weight may provide benefits from a focus on modifying the behaviors of both children and their matched parents [51].

Mackenbach et al. designed a cross-sectional survey embedded in the previously reported Generation R Study, a population-related cohort with data accessible concerning BMI and problem behaviors for 3137 children at the age of 3 to 4 years in the Netherlands [52]. Problem behavior was determined by the Child Behavior Checklist (CBC), and eating patterns were evaluated by the CEBQ. This study showed that children presenting enhanced levels of emotional problems exhibited decreased BMI-SDS after adjusting for related covariates for parent reports of emotional problems. Behavioral problems were not related to BMI. Emotional and behavioral problems were not related to underweight or overweight. The relationship between emotional problems and BMI-SDS diminished to non-significance after adjusting to certain eating behaviors, i.e., they were ascribed to satiety responsiveness, fussiness, and emotional undereating. In this population-based survey, emotional problems in preschool children were inversely associated with BMI, and the above relation was completely supported by food-avoidant eating behaviors [52].

A prospective study explored potential associations of mothers’ feeding practices, children body weight increase and obesogenic eating patterns in children aged 2 years [53]. A sample of 323 mothers and their matched children were enrolled. Mothers completed a questionnaire evaluating parents’ feeding methods and children eating behaviors at baseline and one year later. Children BMI was estimated at both time points. Elevations in children’s BMI z-scores throughout the follow-up period were predicted by mothers’ instrumental feeding methods. Also, constraint, emotional feeding, encouragement to eat, weight-based limitation and fat constraint were related to the development of obesogenic eating behaviors in children, including emotional eating, tendency to overeat and food approach behaviors (like enjoyment of foods and good appetite). Mothers’ monitoring, however, predicted reductions in foods approach eating behaviors. Partial support was also noted for children’s eating behaviors predicting mothers’ feeding methods. In view of the above considerations, maternal feeding practices were considered to exert a crucial impact on the development of body weight increase and obesogenic eating patterns in children and could be possible targets for efficient prevention interventions directed to reduce childhood obesity [53].

Hirsh et al. performed a longitudinal study using the Eating Pattern Inventory for Children (EPI-C), which has not been applied for children at the age of 8 years, exploring potential relationships with body weight [54]. In fact, 521 children of the Ulm Birth Cohort Study (UBCS; age eight) completed the EPI-C and BMI was evaluated. The primary sample included 1045 healthy children (childbirth weight 2000 gr or above) from Germany. Of this primary sample, 634 took part in the 8-year follow-up, performed from March 2009 to May 2010, and of those, 537 were enrolled for additional assessment. MANOVA and cluster analysis found significant correlations of eating patterns with BMI. Moreover, abnormal eating behavior was associated with being overweight. The EPI-C was recognized as an effective measurement tool concerning this children age group. Overweight children consciously restricted their eating. However, the participation rate of the follow-up sample was considerably lower compared to the initial sample. However, response rates of 50% were not unusual in birth cohort studies [54].

A more recent cross-sectional study evaluated differences in children’s eating behavior in relation to their nutritional status, gender and age [55]. Male and female children at the age of 6–10 years from Brazil were enrolled. CEBQ subscales evaluated eating behaviors: Foods Responsiveness, Enjoyment of Foods, Desire to Drink, Emotional Overeating, Emotional Undereating, Satiety Responsiveness, Food Fussiness and Slowness in Eating. The enrolled participants included 335 children with a mean age of 87.9 ± 10.4 months and 49.3% exhibited normal body weight (*n* = 163), 26% were overweight (*n* = 86), 15% were affected by obesity (*n* = 50) and 9.7% were affected by severe obesity (*n* = 32). Children with excessive body weight indicated elevated levels concerning the CEBQ subscales, which were related to “foods approach” and enhanced levels on two “foods avoidance” subscales compared to normal-weight children. Eating behavior was not associated with children’s gender and age [55].

Steinsbekk et al. designed a longitudinal study to evaluate appetite behaviors, physical activity, and television (TV) time as predictors of alteration in BMI Standard Deviation Score (BMI SDS) from age 6 to 8 years and to investigate the impact of BMI SDS (from age 4) on appetite behaviors [56]. In fact, this study enrolled 995 Norwegian children aged 4 years. Moreover, 760 and 687 of the above children participated in the evaluation at the ages 6 and 8 years, respectively. Appetite behaviors were evaluated by the CEBQ, physical activity was determined by accelerometers, and TV time was estimated by parents’ reports. Elevated food responsiveness predicted an increase in BMI SDS. A reversed impact was also noted, as elevated BMI SDS predicted enhanced food responsiveness and decreased satiety responsiveness. BMI SDS was not associated with physical activity levels and TV time. Children’s eating could be especially induced by the sight and smell of foods, which may lead to prospectively elevated body weight increase. Excessive body weight and body weight increase could also predict elevated food approaching behavior [56].

Sanchez et al. performed a cross-sectional study to evaluate the relationship of eating behavior and BMI z-scores in 1058 children aged 7–10 years from Chile [57]. Eating behavior scores were measured through the CEBQ. A substantial association of eating behavior with BMI z-scores of children was noted, revealing that BMI in children aged 7–10 years was directly related to pro-intake eating behavior levels and inversely related to anti-intake eating behavior levels [57].

Another cross-sectional study examined the impact of emotional eating in 5426 children at the age of 9–11 years from 12 countries (Australia, Brazil, Canada, China, Colombia, Finland, India, Kenya, Portugal, South Africa, the United Kingdom, and the United States) and five continents [58]. Emotional eating, food intake, and TV-watching were evaluated, utilizing self-reported questionnaires, and physical activity and nocturnal sleep duration were determined by accelerometers. Emotional eating factor levels were determined by confirmatory factor analysis, and nutritional habits were recognized by principal component analysis. The relationships between emotional eating and health behaviors and BMI z-scores were explored, applying multilevel models containing age, sex, and family income as covariates. Emotional eating was consistently (within 12 survey sites) related to a non-healthy dietary pattern, supporting evidence that the relationship was not limited to Western countries. Positive relationships of emotional eating with physical activity and TV watching were not stable among sites. The above evidence tended to be analogous for boys and girls. Emotional eating was not related to BMI. In view of the above considerations, this study proposed that prospective studies investigate if elevated emotional eating in children may predict the development of no desirable nutritional habits and obesity over time [58].

The purpose of a clinical trial was to assess the validity of the EES-C in a clinical sample of USA children seeking weight-loss treatment [59]. The Emotional Eating Scale—Adapted for Children and Adolescents (EES-C) assessed children’s urge to eat in response to experiences of negative affect. Using a hierarchical bi-factor approach, the validity of the EES-C was evaluated to determine a single general construct, a set of two separate related subconstructs, or a hierarchical prearrangement of two constructs, and explored the consistency in a treatment-requesting overweight or obese children at the age of 8–12 years. Factor-extraction comparison methodologies supported a single primary construct reinforcing EES-C in this clinical sample. The bi-factor indices showed obvious indications that most of the consistent variance in the total score was ascribed to the general construct. After adjustment for potential associations with the primary construction, the remaining associations amongst groups of items did not support further consistent constructions. The above findings supported evidence that the main explanatory importance of the EES-C amongst overweight or obese treatment-requesting children need to be positioned on a single general construct, and not on the 3 or 5 subconstructs, as was earlier reported [59].

Goel et al. designed a cross-sectional study including 400 school-going children aged 11–13 years from India with the aim to estimate the possible associations between dental caries and BMI, perceived stress as well as emotional eating [60]. BMI levels were not shown to be considerably different amongst children with and without caries in first and lasting teeth. Emotional eating was assessed by Emotional Eating Scale (EES). EES score was shown to be considerably elevated amongst caries-free children compared with those who exhibited mean “decayed and filled teeth”/“decayed missing and filled teeth (dft/DMFT)” score > 0 [60].

### 3.3. Association of Mental Disorders with Children’s Emotional Eating

There are several clinical studies evaluating the relationships between diverse mental disorders and emotional eating. All relevant clinical studies are included in Table 3 [61,62,63,64,65,66,67,68]. More to the point, a clinical survey explored potential relationships of sleep onset latency with emotional eating in a minority sample of children [61]. This cross-sectional school-related survey of sleep, psychological constructions, nutrition and physical activity was performed in six public and private schools in Los Angeles County. An ethnically diverse sample of 356 third through fifth graders produced private self-reported questionnaires. Multilevel regression (MLM) analyses were applied for evaluating potential relationships after adjustment for sex, nationality, and the random impact of school. Girls were 57% of the overall sample, which mainly comprised Latino (42.6%), followed by African Americans (21.6%) and Asians (19.2%). MLM showed that there were considerable relationships of sleep onset latency and emotional eating, depressive symptomology and trait anxiety. Sobel’s criterion for mediation indicated that trait anxiety but not depression symptoms was a mediator of the association of sleep onset latency with emotional eating. The above findings provided a mechanism by which sleep onset latency may be associated with emotional eating. The above evidence also suggested that sleep onset latency may be related to increased anxiety, depression symptomatology, and emotional eating. However, causal effects cannot be derived due to the cross-sectional design of this study. In this aspect, the authors suggested that additional studies are required to investigate the likelihood that difficulties falling asleep may result in emotional dysregulation that in turn results in emotional eating. Moreover, emotional eating could be considered as an alternative strategy by which sleep disorders may result in overweight and obesity [61].

Psychological stress has been suggested to change dietary pattern towards more non-healthy choices and as such to contribute to overweight or obesity. The interrelationships among stress, emotional eating behavior and nutritional habits have not often been examined in young children. Nonetheless, investigation in children is crucial as the impact of nutritional behavior is established beginning from childhood and could persist into adulthood. In this aspect, in 437 children aged from 5 to 12 years of the ChiBS survey, in Belgium, stress was determined via questionnaires concerning stressful measures, emotions (happy, angry, sad, anxious) and difficulties (emotional, peer, conduct and hyperactivity) [62]. This study used the DEBQ, which can evaluate three types of eating behavior. For this cross-sectional survey, only emotional eating (eating in response to harmful emotions) could be taken into consideration. Data concerning children’s emotional eating patterns and nutritional habits was retrieved: incidence of fatty foodstuffs, sweet foodstuffs, snacks (fat and sweet), fruits and vegetables. Stressful episodes, harmful emotions and difficulties were directly related to emotional eating. Positive relationships were detected among difficulties and both sweet and fatty foodstuff intake. Inverse relationships were noted among stressful episodes and fruit and vegetable intake. Collectively, stress was correlated with emotional eating and no healthier nutritional habits and may therefore influence the development of overweight or obesity in children. However, emotional eating behavior was not found to facilitate the stress–diet relationship [62].

Another cross-sectional design aimed to explore whether stressful biomarkers and negative emotionality could be associated with emotional eating and to understand the impact of parents’ behavior in the above association [63]. The study population included 476 children at the age of 2 to 6 years from the Swiss cohort survey SPLASHY. This study evaluated the children’s emotional eating, negative emotionality and parents’ behavior based on parents’ reports. To explore cortisol and salivary alpha-amylase concentrations, salivary samples were assembled for 2 days. Based on the overall sample of children, 1.1% indicated emotional overeating and 32.9% emotional undereating. Negative emotionality was associated with emotional overeating and emotional undereating. There was no relationship between stress biomarkers and emotional eating. The enrolled parents more frequently reported emotional undereating compared to emotional overeating of their children. Moreover, both of them were associated with temperament. This study concluded that a specific subgroup of children with problematic temperament may have an increased risk of eating and body weight regulation difficulties in later childhood; however, stress may not exert any impact on the above conditions [63].

Sheinbein et al. also designed a cross-sectional study to evaluate the incidence and the related agents of depressive and anxiety symptomatology of treatment-searching overweight and obese children [64]. Two hundred forty-one children aged 7–11 years from the USA and their matched parents were subjected to assessments prior to the start of family-related behavioral body weight loss treatment. A social–ecological model was applied as a framework for analyzing potential agents related to depressive and anxiety symptomatology. Amongst the study population, 39.8% (96/241) children met criteria for depressive and/or anxiety symptoms. Parents completed the Child Behavior Checklist, a comprehensive evaluation of children’s behavioral and emotional functionality that has shown high validity and consistency. Children eating disease pathology, parents’ utilization of psychological control (i.e., a parent behavior style categorized by emotional manipulation), and reduced child personal social condition were substantially related to elevated child depression symptoms. Children’s eating disease pathology and parents’ psychological monitoring were substantially related to elevated children anxiety symptoms. In total, about 40% of children showed psychopathological symptomatology, and a variety of correlations were noted. Hence, children’s body weight reduction providers could take into consideration screening and addressing mental health concerns (and related factors) prior to and during treatment [64].

Another cross-sectional study aimed to explore whether comorbid anxiety and depression in eating disorders in children may influence the association between stress in children and their emotional eating behaviors [65]. This study enrolled 1120 children aged 11–12 years from 60 public primary schools across Taiwan. Eating Disordered Scale and DEBQ were used for the assessment of emotional eating. This study showed that anxiety and depressive levels substantially mediated the association of stressful behavior with emotional eating levels. Particularly, those children who self-reported elevated anxiety and depressive levels exhibited increased stressful behavior and emotional eating compared with those with lower anxiety and depressive levels. The above results suggested that when an eating disorder may be related to stressful behavior and emotional eating, the co-concurrence of anxiety and depression could enhance the probability of emotional eating [65].

Food insecurity may increase the risk of childhood obesity; however, the mechanisms explaining this relationship have not elucidated yet. On the other hand, it has been well-recognized that parents experience greater psychosocial stress in the context of food insecurity. Under these conditions, children from food-insecure households could have diverse appetite behaviors. In this respect, a cross-sectional study examined potential relationships between food insecurity and appetitive behaviors in children aged 3–5 years from the USA and explored if social, emotional and structural properties of the home conditions may attenuate the above association [66]. In a low-income sample of 504 parents and their matched children, parents completed the household food security module and the CEBQ. A subsample (*n* = 361) self-reported perceived stress, depression symptomatology, household confusion and family functionality. Children were classified as food-secure, household food-insecure and children food-insecure. Food responsiveness and emotional overeating were elevated amongst children in the children food insecurity group compared with the food-secure grouping. Children food insecurity was only related to greater food responsiveness amongst children of parents stating elevated scores of perceived stressful behaviors and decreased scores of family functionality. No differentiations were noted in food responsiveness by food security status at mean or decreased scores of perceived stressful behaviors or at mean or elevated scores of family functionality. The above findings supported evidence that child food insecurity could increase the probability of developing obesity via differences in appetite behaviors. Moreover, concerning low-income families, stress control and enhancing family dynamics could be crucial agents for interventional surveys designed to enhance child appetite behavior [66].

Feeding difficulties are widespread at the first stages of children’s lives, and certain surveys have suggested that feeding difficulties could be related to psychopathological states. Some longitudinal surveys have investigated if toddler feeding difficulties predict psychopathological states [67]. For this purpose, 1136 children and their matched mothers from the Upstate KIDS cohort survey in the USA provided records for children aged 2.5 and 8 years. Food refusal (picky eating) and mechanical/distress feeding difficulties and developmental delays were evaluated at 2.5 years. Children eating behaviors (enjoyment of foods, food fussiness, and emotional undereating and overeating) and children psychopathological states (attention-deficit/hyperactivity (ADHD), oppositional-defiant (OD), conduct disorder (CD), and anxiety/depression) symptomatology were also evaluated at 8 years [67]. Mechanical/distress feeding difficulties at age 2.5, but not food refusal difficulties, were associated with ADHD, problematic behavior (OD/CD), and anxiety/depression symptomatology at 8 years in models after adjustment for eating behaviors at 8 years and children and family covariates. Relationships amongst mechanical/distress feeding difficulties were elevated for ADHD and problematic behavior compared to anxiety/depression symptomatology, though all were moderate. Model estimates were analogous for both boys and girls. In this respect, this survey suggested that early mechanical and mealtime distress difficulties could be considered as greater predictors of later psychopathological states compared to food refusal. Moreover, parents and pediatricians could monitor children with mechanical/distress feeding problems for signs of developing psychopathology [67].

Doğan Güney et al. designed a cross-sectional study to assess emotional eating and perceived stressful conditions with mindfulness in 349 Turkish children aged 9–11 years [68]. The Mindfulness Scale for Children (BAU-MSC), the Emotional Eating Scale for Children and Adolescents (EES-C), and the Perceived Stress Scale in Children (8–11 years) (PSS-C) were used. A positive, weak relationship was found between children’ age and body weight and height. A positive, weak relationship was also observed between children’ age and emotional eating, anxiety–anger–disappointment subscales and perceived stress scores. Significant differences were also noted in terms of BMI and the availability of regular medication [68].

### 3.4. Associations of Children’s Dietary Habits with Emotional Eating

Psychological stress has been considered to alter nutritional habits towards more unhealthy choices and as such to lead to overweight. Emotional eating behavior may be a mediating mechanism, as reported in Table 4 [69,70,71,72,73,74,75]. Research in children has been considered as crucial, as the foundations of dietary nutritional patterns may be established beginning from childhood and could persist in adulthood. In this aspect, a longitudinal study aimed to explore if eating behavior characteristics may have continuity and stability during childhood [69]. More to the point, mothers of 428 twin children from the Twins Early Development Study joined a survey of eating and weight in 1999 when the children were 4 years old. Families communicated once more in 2006 when the children were aged 10 years, with provided data on 322 children. At both times, mothers completed the CEBQ for each child. Continuity was associated between scores at the two time points, and stability by alterations in mean scores over time. For all CEBQ subscales, associations between the two time-points were highly considerable. For satiety responsiveness, eating slowness, food responsiveness, food enjoyment, emotional overeating and food fussiness, associations ranged from r = 0.44 to 0.55, with lower continuity for emotional undereating (r = 0.29). Over time, satiety responsiveness, eating slowness, food fussiness, and emotional undereating were reduced, and food responsiveness, food enjoyment and emotional overeating were elevated. Eating patterns, containing those associated with a tendency to overeat, arose early in the development path and indicated levels of personal continuity analogous to constant personality characteristics. Appetite characteristics associated with increased satiety decreased with maturation, and those associated with food responsiveness were enhanced. Hence, the above study supported evidence that this pattern may be reliable with strong tracking of BMI combined with an advancing rise in the probability of obesity [69].

A case–control study was designed to explore the relationships between preschool children’s emotional eating and parents’ feeding methods by applying an experimental strategy of children’s mood and food consumption in UK [70]. Twenty-five 3- to 5-year-old children and their matched mothers were assembled simultaneously and consumed a typical meal to satiety. Mothers filled out questionnaires concerning their feeding methods. Children were enrolled to a control or harmful mood state, and their intake of snack foods in the absence of hunger was evaluated. The emotion rating scale consisted of a series of five stylized, non-gender-specific faces ranging from widely smiling (to show happiness) to a maximum down-turned mouth (to show unhappiness). This was applying for evaluating the emotional eating of children. Children of mothers who frequently utilized foods to control emotions consumed more cookies in the absence of hunger compared to children of mothers who utilized this feeding method rarely and independently of any state. Children of mothers who frequently utilized foods for emotional modulation reasons frequently consumed chocolate in the experimental conditions compared to control conditions. The above pattern was inverted for children of mothers who did not tend to use food for emotional regulation. However, there were no significant effects of maternal use of restriction, pressure to eat, and use of foods as a reward on children’s snack food consumption. Hence, children of mothers who use food for emotional regulation may consume more sweet palatable foods in the absence of hunger than children of mothers who may use this feeding practice infrequently. Overall, emotional overeating behavior could happen concerning the negative mood in children of mothers who utilize foods for emotional modulation reasons [70].

A longitudinal survey was designed to explore the relationships among two kinds of emotion regulation (reactivity and inhibition) and two kinds of non-hunger-based eating (emotional eating and external eating) [71]. For this purpose, 782 rural secondnd graders were followed up through third grade. More to the point, children took part in one-on-one interviews with qualified personnel at schools utilizing the Children’s Emotion Management Scales and the revised DEBQ. This survey showed that children’s emotional modulation was substantially related to external and emotional eating among grades. Reactivity was more highly associated with eating modulation than inhibition. Regression analysis indicated that second to third graders increased in external and emotional eating, which were predicted by elevations in reactivity to anger and reactivity to fear. Taking into consideration the established relationships in former research among low behavior modulation and obesity during childhood, the evidence derived by the present survey linked children’s emotional reactivity, and emotional and external eating (both forms of behavior dysregulation) were crucial for updating prevention and treatment strategies. According to the above evidence, targeting children emotional regulation in addition to behavior regulatory skills as part of prevention and interventional strategies could improve program efficacy [71].

In a case–control study, the relationship between parent reports of children’s emotional overeating and emotional undereating applying CEBQ and children’s eating behavior during circumstances of harmful emotions was investigated [72]. Sixty-two mothers with children aged from 34 to 59 months consumed a meal to satiety. Children were randomly enrolled to harmful mood induction or neutral conditions. Children had access to snack foods for 4 min and their intake was determined. Adjusting for covariates, children who were rated as higher in emotional undereating on the CEBQ ate fewer kilocalories from crisps/potato chips and cookies when in a negative mood state, but not when in a neutral mood. There were no significant relationships between maternal levels of emotional overeating on the CEBQ and children’s snack consumption in both conditions. The above survey provided evidence that the CEBQ had high validity concerning the emotional undereating scale in children aged from 3 to 5 years. Additional research, containing induction of various mood conditions, should be performed to examine if the emotional overeating scale may actually capture young children’s emotional overeating [72].

Steinsbekk et al. performed a longitudinal study to explore whether temperament may be implicated in the etiology of eating behaviors during childhood [73]. In fact, 997 Norwegian children were followed up from 4 to 10 years. A temperament of harmful affectivity, effortful monitoring, and surgency were determined through CBEQ. The CEBQ examined four ‘foods approach’ behaviors (‘foods responsiveness’, ‘foods’ enjoyment, ‘emotional overeating’, ‘drink desire’) and four ‘foods avoidant’ behaviors (‘emotional undereating’, ‘satiety responsiveness’, ‘foods fussiness’, ‘eating slowness’). The longitudinal associations of temperament with eating behaviors were evaluated after adjusting for all unmeasured time-invariant factors. After adjustment for confounding factors, elevated harmful affectivity predicted better ‘foods approach’ and ‘foods avoidant’ behaviors, as did decreased effortful regulation. Elevated surgency was longitudinally associated with additional ‘foods approach’ and lower ‘foods avoidant’ behaviors, however, only at some ages and with the exception of exclusion of emotional overeating and undereating. These results indicated that temperament may be implicated in the etiology of children’s eating behaviors. Thus, harmful affectivity could influence both ‘foods approach’ and ‘foods avoidant’ behaviors. Because children are prone to react, presenting harmful effects that may exhibit a high probability of obesogenic and disturbed eating behavior, their mothers and fathers should predominantly be informed of how to support healthy eating [73].

A cross-sectional study was conducted on 178 children aged 8–9 years in Italy [74]. In this study, relevant questionnaires were used on both children and their matched mothers, aiming at exploring potential factors which may be related to emotional eating as behavioral characteristics or compliance to the Mediterranean diet. Emotional undereating and emotional overeating were determined by utilizing two subscales (with four questions each) of the CEBQ. Emotional undereating was directly related to emotional symptoms. Emotional overeating was positively related to both emotional symptoms and hyperactivity and inversely associated with peer problems. Emotional undereating was also directly related to the number of siblings and inversely correlated with high Mediterranean diet compliance. Thus, this study supported evidence that children’s emotional eating was related to both nutritional habits and behavioral characteristics [74].

An et al. designed a cross-sectional study to evaluate the incidence of ultra-processed food intake in 408 children aged from 6 to 36 months and recognize its relationships with caregivers’ emotions and instrumental feeding, and children’s emotional eating assessed by the CEBQ [75]. Caregivers’ emotional and instrumental feeding was directly related to children’s intake of ultra-processed foods, an elevated incidence of ultra-processed foods consumption weekly, and a larger amount of ultra-processed foods consumption weekly. Children’s elevated incidence of emotional undereating was related to their ultra-processed foods intake and a higher frequency of ultra-processed foods consumption weekly. Children’s emotional undereating was substantially implicated within the relationships among caregivers’ emotions and instrumental feeding and children’s consumption of reconstituted meat products. Caregivers should be educated to avoid emotional and instrumental feeding practices and cultivate children’s good eating habits to improve children’s diet quality [75].

### 3.5. Other Factors Influencing Children’s Emotional Eating

There are several other factors that can be related to the emotional eating of children, as depicted in Table 5 [76,77,78,79,80,81]. In this respect, leptin is mostly released from adipose tissue and functions in the hypothalamus to monitor energy intake. The existence of mutations in the gene or the receptor of leptin may induce monogenic obesity; however, this event is rather scarce. On the other hand, leptin and leptin receptor polymorphisms have been related to obesity, which has been ascribed to the relationship between body weight and eating behaviors. In this aspect, a study explored the relationship of leptin and leptin receptor polymorphisms with childhood obesity and eating behavior [76]. In fact, 221 Chilean children affected by obesity with a mean age of 9.7 years were enrolled in a cross-sectional study. The parents of 134 of these children were also enrolled to examine the relationship of leptin and leptin receptor polymorphisms with obesity. Eating behaviors were determined by the questionnaire of three factors progenitors’ version (TFEQ-P19) and eating behavior in children (CEBQ). No substantial difference among the explored polymorphisms and childhood obesity, after adjusting for multiple comparisons, was noted. The dimensions “Slow eating”, “emotional eating”, “foods’ enjoyment” and “uncontrolling eating” were considerably related to some polymorphisms of leptin and leptin receptor. These findings supported evidence that there could be a relationship of polymorphisms of leptin the and leptin receptor genes with eating behaviors in Chilean children affected by obesity [76].

Another study aimed to explore the impact of leptin in the relationship between stress and emotional eating as assessed in primary school children [77]. In a two-stage prospective survey with 308 Belgium children aged 5–12 years, the relationships of fasting serum leptin with stated stress (harmful events and emotional difficulties) were explored. Stress was assessed by salivary cortisol (total cortisol output and awakening response). Additionally, the relationship of fasting serum leptin with emotional eating and food consumption frequency was evaluated. Analyses were split by gender. Children completed the DEBQ concerning their usual eating behavior. Mediation and moderation by leptin change were assessed. This study showed that the stress marker (overall cortisol output) was substantially related to elevated leptin concentrations, however, only in females and cross-sectionally. Only in boys were leptin levels correlated with reduced emotional eating. Leptin was not identified as a considerable predictor of non-healthy food intake. Leptin alteration did not appear to act as a mediator factor but as an enhancing moderator within the relationship of stress (high cortisol output and emotional difficulties) and emotional eating in females [77].

Furthermore, the Gemini twin birth cohort provided data from 2054 five-year-old children, containing parents’ scores of emotional overeating and emotional undereating using the CEBQ [78]. Genetic and environmental impacts on variation and covariation in emotional undereating and emotional overeating were recognized utilizing a bivariate twin model. Variation in both behaviors was highly supported by traits of the environment completely common to the twin pairs. Genetic influence was low. Emotional overeating and emotional undereating were positively interrelated, and this relationship was clarified by common shared environmental influences. Many of the shared environmental impacts related to emotional undereating and emotional overeating were similar. Childhood emotional overeating and emotional undereating were etiologically different. Moreover, the trend to consume more or less in response to emotions was shown to be learned rather than inherited [78].

A longitudinal study explored the relative genetic and environmental impacts on emotional overeating in toddlerhood and young childhood. Data were derived by Gemini, a population-based cohort of 2402 British twins born in 2007 [79]. Emotional overeating was measured using the “emotional overeating” scale of the CEBQ for 16 months and 5 years. A longitudinal quantitative genetic model established that genetic influences on emotional overeating were minimal. On the other hand, shared environmental influences explained most of the variance. Emotional overeating was moderately stable from 16 months to 5 years and maintaining environmental agents allocated by twin pairs at both ages supported the etiological relationship [79].

Additionally, data from the previous British twin cohort indicated that environmental rather than genetic agents influence characteristic alterations in both behaviors in youth childhood. The above survey aimed to reproduce the previous findings in a subsample (*n* = 394) of 4-year-old twins enrolled from high or low probability of developing obesity from another population-based cohort of British twins (the Twins Early Development Study) [80]. Parents’ scores of children’s emotional overeating and emotional undereating were analyzed utilizing genetic modeling settings. The genetic impact was not substantial, and combined environmental agents described 71% of the variance in emotional overeating and 77% in emotional undereating. The two behaviors were directly related to each other, and about two-thirds of the shared environmental agents affecting emotional overeating and emotional undereating were similar. Thus, children’s emotional eating may be influenced by the home family environment, and parents are therefore promising intervention targets [80].

Stress-induced emotional eating has commonly been considered as a probability agent of being overweight or obese. Earlier research has shown that both the human serotonin transporter gene (5-HTTLPR) and children’s reactive temperament could be crucial candidates to contribute to the elucidation of individual differences in stress-induced emotional eating and weight. In this aspect, another cross-sectional survey investigated the potential indirect impact of genetic and environmental susceptibility (i.e., the interaction of 5-HTTLPR with reactive temperament) on body weight (as measured by body fat percentage) facilitated by stress-stimulated emotional eating [81]. One hundred and forty-seven children aged from 4 to 6 years from the USA, along with their primary caregiver, completed laboratory tasks and questionnaires, which evaluated children’s reactive temperament, stress-stimulated emotional eating, and body fat percentage. The interaction of 5-HTTLPR with impulsivity and harmful affectivity substantially predicted body fat percentage. The interaction of 5-HTTLPR and impulsivity and harmful affectivity also considerably predicted both overall calorie intake and rate of overall calorie intake. Nonetheless, the mediation aspect of this statistical model was not proved. Thus, children’s reactive temperament could be a critical indicator of how children approach eating when stressed. Moreover, mental health providers may consider recommending policies to decrease emotional eating among children with the short allele variant and moderate to high impulsivity as well as children presenting long allele variant and high negative affectivity [81].

## 4. Discussion

Based on a thorough search of the most accurate international scientific databases, we retrieved 65 clinical studies exploring the impact of emotional eating in child populations. Most of them (*n* = 24) assessed the parental behavior effect on children’s emotional eating. A considerable number of clinical studies (*n* = 18) evaluated the association of overweight/obesity and emotional eating in children. Moreover, eight clinical studies evaluated the association of mental disorders with children’s emotional eating and another nine clinical studies evaluated the association of children’s dietary habits with emotional eating. The remaining six clinical studies evaluated the association of other factors with the emotional eating of children. Almost half of the clinical studies (*n* = 30) have been performed in the USA (*n* = 16) and the UK (*n* = 14).

The vast majority of clinical studies (*n* = 52) have a cross-sectional design, which cannot support causality effects. However, most of them include an adequate number of enrolled children and parents. Certain clinical studies (*n* = 18) have a prospective design and an adequate number of enrolled children and parents, supporting causality effects. A small number of clinical studies (*n* = 4) have a case–control design with a rather small number of assigned children and parents, except for one study. More than half of the retrieved clinical studies (*n* = 35) used the well-recognized CEBQ tool to assess the emotional eating of children, while DEBQ-C was the second most frequently used tool. The remaining clinical studies used diverse questionnaire tools to assess emotional eating, increasing the heterogeneity of the questionnaire tools and reducing the overall quality of the derived results and conclusions. In this respect, CEBQ seems the most appropriate tool to assess children’s emotional eating, highlighting its use in relevant future studies.

Taking into consideration the existing literature evidence, there are four distinct risk factors that may promote the risk of developing emotional eating in children, Firstly, certain parental behaviors may increase the risk of developing emotional eating of their children. Unusual parental eating behavior was highly related to an adverse parent–child relationship independently of children’s body weight. Maternal symptoms of depression, anxiety, and stress may lead to maternal emotional eating, which could increase the risk of developing child emotional eating. Moreover, children’s emotional eating seems to be attributed to interrelationships between greater parent emotional eating, the use of food as a reward, restriction of food for health reasons and negative affective temperaments. Secondly, children diagnosed with emotional eating appear to have a higher risk of developing overweight or obesity. On the other hand, overweight and obese children seem to be characterized by higher BMI. Thirdly, children’s stressful events, negative emotions and problems seem to be associated with a higher risk of emotional eating and an un-healthier dietary pattern. In addition, children with higher anxiety and depression scores may have a higher risk of stress experience and emotional eating. Fourthly, children diagnosed with emotional eating appear to consume more unhealthy foods such as sweet palatable foods as well as ultra-processed foods in the absence of hunger.

Although the currently available clinical studies revealed that emotional eating is gradually increasing in children, there are certain limitations that should be considered. More to the point, most studies have a cross-sectional design, which cannot support a causality effect, highlighting the need to perform more longitudinal studies. In addition, several studies did not concern potential confounding factors that may exert an impact on the emotional eating of children. There is also heterogeneity among the available studies concerning the questionnaire tools that they used to assess children’s emotional eating. Moreover, most of the studies are based on self-reported data, which increases the risk of potential recall biases. Lastly, there is heterogeneity among the available studies concerning the age range of enrolled children. Some studies are focused on pre-school children, while others are focused on pre-adolescent children. The above fact renders the direct comparison of these studies unreliable, as the first stages of children’s lives are characterized by different dietary requirements and guidelines, e.g., 2–5-year-old children compared to 9–12-year-old children.

## 5. Conclusions

The currently existing clinical evidence reveals that emotional eating, and especially emotional overeating, is gradually increasing at the first stage of human life, leading to overweight and obesity in the child population. Unhealthy dietary practices of parents and the increased prevalence of mental disorders in children may trigger the development of emotional eating even at the first stage of human life. However, the currently available clinical evidence has mainly been derived from cross-sectional studies, which cannot support the causality effect. In this respect, clinical studies with a longitudinal design and adequate follow-up are highly recommended in order for more reliable conclusions to be drawn. Systematic public policies should be developed to educate parents to recognize whether their children are at risk of emotional eating and to adopt healthy dietary patterns. Moreover, educational seminars should be incorporated into the school communities to directly educate children for the potential benefits of healthy dietary patterns.

## Figures and Tables

**Table 1:** Clinical studies assessing the impact of parental behavior on children’s emotional eating.

Study Type	Study Population	Children EE Assessment *	Basic Results	References
Cross-sectional study	75 USA children (40 boys and 35 girls) aged 3–6 years	Behavioral index of disinhibited eating	Familial effects on childhood overweight may be different based on parent and child gender (*p* < 0.05). Mothers’ dietary disinhibition may mediate familial similarities in degree of overweight for mothers and daughters (*p* < 0.05).	Cutting et al., 1999 [19]
Cross-sectional study	368 Germany children (164 boys and 204 girls) aged 8–11 years (95.7% aged 10–11 years)	Not validated questionnaire	Unusual eating attitude was highly related to a harmful parent–child relationship regardless of childhood body weight (*p* < 0.001).	Schuetzmann et al., 2008 [20]
Cross-sectional study	450 USA children aged 6–8 years (gender not reported)	DEBQ-C	Emotional eating was adversely predicted by reliable parental behavior (*p* < 0.036) and family open affection (*p* < 0.001) and emotional expression (*p* < 0.001) and directly predicted by parents’ reduced response to childhood harmful emotional behavior (*p* = 0.003).	Topham et al., 2011 [21]
Longitudinal study	1327 Denmark children aged 5–7 years (full text and gender cannot be retrieved)	A composite instrument assessing eating behaviors and their impact	Picky consumption was related to psychopathology within diseases (*p* < 0.001). Emotional undereating was related to emotion and functional somatic symptomatology (*p* < 0.001).	Micali et al., 2011 [22]
Cross-sectional study	106 USA children (45.3% boys and 54.7% girls) aged 8–12 years	CEBQ	Parent variables were more intensely correlated with childhood emotional eating, controlling for child age and sex (*p* < 0.001). Emotional eating attitude was the most significant parent factor related to children’s emotional eating (*p* < 0.001).	Braden et al., 2014 [23]
Cross-sectional study	288 Belgium children (145 boys and 143 girls) aged 6–12 years	DEBQ-C	A borderline positive relationship was found between sweet food consumption incidence and “coercive control” (*p* = 0.014) and a marginal negative correlation between fruit and vegetable consumption incidence and “overprotection” was noted (*p* = 0.102 and *p* = 0.009, respectively). Children more commonly consumed soft drinks after their parents had decreased levels of “structure” and increased levels of “overprotection” (*p* = 0.123 and *p* = 0.031, respectively).	Philips et al., 2014 [24]
Cross-sectional study	306 Australian children aged 2 years (gender not reported)	DEBQ-C	Mothers’ and children’s emotional eating was related to mother’s symptomatology of depression, anxiety, and stress (*p* < 0.001 and *p* < 0.05, respectively).	Rodgers et al., 2014 [25]
Cross-sectional study	95 USA children (49 boys and 46 girls) aged 4.5–9 years	DEBQ-C	The relationship of parental and childhood emotional eating was mediated by feeding for emotional regulation when children’s self-regulation concerning eating was decreased (*p* = 0.001), but not when self-regulation in eating was increased (*p* = 0.105).	Tan et al., 2015 [26]
Longitudinal study	35 UK children (16 boys and 19 girls) initially 4–5 years and finally aged 5–7 years	Not validated questionnaire	Parents excessively regulate children’s foodstuff consumption and seem to accidentally educate their child to highly consume palatable foods to manage harmful emotional behavior after a 2-year follow-up (*p* < 0.001).	Farrow et al., 2015 [27]
Cross-sectional study	77 UK children (49.0% boys and 51.0% girls) aged 3–12 years	CEBQ	A considerable direct effect of maternal attachment anxiety on child emotional overeating was noted (*p* = 0.002). A substantial indirect impact of mothers’ anxiety attachment on children’s emotion over-eating through emotion feeding approaches (*p* = 0.02). A considerable indirect impact of mothers’ attachment anxiety on emotion eating approaches through children emotional overeating (*p* = 0.01).	Hardman et al., 2016 [28]
Cross-sectional study	60 Germany children (27 boys and 33 girls) aged 18–55 months	CEBQ	An indirect impact of mentalization through emotional eating on mothers’ (*p* < 0.05) but not on children’s weight and through mother–child attachment on children’s weight (*p* = 0.45 and *p* = 0.42).	Keitel-Korndörfer et al., 2016 [29]
Longitudinal study	229 UK children (120 boys and 129 girls) with a mean age of 8.73 years at borderline and a one year follow-up	EPIC	Perceptions of parents’ force to eat and limitations considerably diminished the relations among eating behaviors over a 12-month period (*p* < 0.001).	Houldcroft et al., 2016 [30]
Cross-sectional study	254 USA children (31.7% boys and 68.3% girls) with a mean age of 4.17 years	CEBQ	The association of parents’ practice of food as a reward and children emotional overeating was partly mediated by children’s self-modulation in consumption after adjusting for parental and child sex, family salary, and race/ethnicity (*p* ≤ 0.001).	Powell et al., 2017 [31]
Cross-sectional study	100 children (33.0% boys and 57.0% girls) aged 8 to 13 years from Switzerland	DEBQ-C	Parents’ critique and, to a lower extent, parents’ emotional overinvolvement were certainly correlated with child emotional intake, and this association was facilitated by children’s harmful pressure (*p* < 0.05).	Munsch et al., 2017 [32]
Longitudinal study	997 Norwegian children (49.1% boys and 50.9% girls) aged 4 years old followed up at ages 6 (795 children), 8 (699 children), and 10 (702 children) years	CEBQ	The elevated amounts of emotion feeding were associated with increased amounts of emotional consumption and vice versa, after adjustment for BMI, and firstly, amounts of feeding and eating (*p* < 0.001). Elevated amounts of temperamental harmful affectivity (at the age of 4 years) enhanced the probability of developing emotional eating and feeding in the future (*p* < 0.001).	Steinsbekk et al., 2018 [33]
Longitudinal study	3514 children (49.1% boys and 50.9% girls) aged 4 years with a follow-up at the age of 10 years from Netherlands	CEBQ	Three patterns of emotional overeating and 5 patterns of food responsiveness were recognized. Obesogenic eating attitude patterns were related to an increased childbirth body weight and BMI, emotional and behavioral problems, mothers’ overweight or obesity and monitoring feeding approaches (*p* < 0.001).	Derk et al., 2019 [34]
Longitudinal study	Norwegian children (49.8% boys and 50.2% girls) followed up at the age of 6 (797 children), 8 (699 children) and 10 (702 children) years	CEBQ	Low (temperamental) soothability and less parental structuring at age 6 were associated with elevated emotional overeating at the age of 10 years (*p* = 0.003) and that decreased family performance at the age of 6 years was associated with higher emotional undereating throughout the same interval (*p* = 0.014).	Bjørklund et al., 2019 [35]
Case–control study	440 children (272 boys and 168 girls) aged 3–6 years from India	CEBQ	A positive association of food avoidance subscales of CEBQ along with certain food-approaching subscales with tooth decay conditions was noted (*p* < 0.01). Parents’ feeding behaviors like encouragement and instrumental feeding resulted in a reduction in children’s tooth decay conditions in comparison to control and emotion feeding (*p* < 0.01).	Nembhwani and Winnier, 2020 [36]
Cross-sectional study	478 Australian children (48.2% boys and 51.8% girls) aged 5–10 years	CEBQ	Maternal emotional overeating and food responsiveness were each positively related to the parallel childhood eating behavior (*p* < 0.01). Both the relation between mothers and childhood emotion overeating and between mothers and childhood food responsiveness were partly facilitated using feeding as a reward and overt limitation (*p* < 0.01).	Miller et al., 2020 [37]
Cross-sectional study	284 USA children (47.2% boys and 52.8% girls) aged 4–6 years	CEBQ	Parents reporting their parenting stress increased, and elevated parenting stress, which was related to more frequent stress to eat and lower frequency of controlling their children’s nutritional behavior (*p* = 0.01 and *p* = 0.03, respectively).	González et al., 2022 [38]
Cross-sectional study	244 UK children (48.0% boys and 52.0% girls) aged 3–5-year	CEBQ	Children’s emotional eating was ascribed to interrelations among higher emotional eating by parents, the usage of food as a reward, constraint of food for health purposes and harmful affective temperaments (*p* ≤ 0.0001).	Stone et al., 2022a [39]
Cross-sectional study	185 UK children (48.0% boys and 52.0% girls) aged 3–5 years	CEBQ	The relationship between maternal reports of maternal emotional eating and child emotional eating was mediated by maternal usage of food as a reward or of restraint for health reasons (*p* = 0.004 and *p* < 0.001, respectively).	Stone et al., 2022b [40]
A laboratory-based experimental study	47 USA (50.0% boys and 50% girls) children aged 3–5 years	Not validated questionnaire	Mothers within both groups who reported elevated emotional eating functioned themselves (*p* = 0.014) and their children (*p* = 0.007) lower amounts of foods, and mothers ate lower food amounts (*p* = 0.045).	Warnick et al., 2022 [41]
Cross-sectional study	2038 children (1001 boys and 1037 girls) aged 10–11 years from Taiwan	Emotional Eating items from the TFEQ-R18 scale	Mothers’ foreign nationality affected children emotional eating mainly by enhancing rewarding (*p* = 0.001) and pressure-to-eat strategies (*p* = 0.01) combined with decreased health literacy (*p* < 0.0001) that eventually lowered management strategies.	Chen et al., 2025 [42]

* DEBQ-C: Dutch Eating Behavior Questionnaire for Children, CEBQ: Child Eating Behavior Questionnaire, EPIC: Eating Patten Inventory for Children, TFEQ-R18: Three-Factor Eating Questionnaire-Revised 18.

**Table 2:** Clinical studies assessing the impact of overweight and obesity on children’s emotional eating.

Study Type	Study Population	Children EE Assessment *	Basic Results	References
Cross-sectional study	292 obese children (40.0% boys and 60.0% girls) aged 9–11 years from Belgium	PCSC	Significant associations were noted between emotional eating and negative feelings of physical competency; between external eating and negative feelings of self-worth; and between both eating styles and diverse dimensions of problematic behaviors (*p* < 0.001 for all).	Braet et al., 1997 [43]
Cross-sectional study	1213 black girls and 1166 white girls at the age of 9 to 10 years from USA	EIES	Black girls had considerably elevated emotion-induced eating scores compared to white girls (*p* < 0.001). In all races, a negative relationship was noted among BMI and emotion-induced eating (*p* < 0.001).	Striegel-Moore et al., 1999 [44]
Cross-sectional study	240 Portuguese children (117 boys and 123 girls) aged 3–13 years (mean age 7.9 years)	CEBQ	All CEBQ sub-scales were substantially associated with BMI z-scores (*p* < 0.001). Food approach scales were also positively associated with BMI z-scores and food avoidance negatively related (*p* < 0.001).	Viana et al., 2008 [45]
Cross-sectional study	135 children (68 boys and 67 girls) aged 6–7 years from Netherlands	CEBQ	BMI z-scores were directly related to the ‘foods approaches’ subscales of the CEBQ (*p* = 0.016, and *p* = 0.027) and negatively with ‘foods’ avoidant’ subscales (*p* = 0.006).	Sleddens et al., 2008 [46]
Cross-sectional study	406 UK children aged 7–9 years (239 children, 51.0% boys and 49.0% girls) and 9–12 years old (167 children, 39.5 boys and 60.5% girls)	CEBQ	Satiety responsiveness/slowness in eating and food fussiness indicated an ordered negative relation to body weight (*p* < 0.0001, and *p* = 0.023, respectively). Food responsiveness, enjoyment of foods, emotional overeating and desire to drink were positively interrelated (*p* < 0.001).	Webber et al., 2009 [47]
Longitudinal study	UK children (boys:girls ratio = 1:1), time points: 6 weeks (811 children), 12 months (620 children), 5–6 years (506 children), 6–8 years (583 children)	CEBQ	Children with elevated emotional overeating and desire to drink presented increased BMIs, while children with higher levels of satiety responsiveness exhibited lower BMIs (*p* < 0.005).	Parkinson et al., 2010 [48]
Cross-sectional study	241 UK children (55.0% boys and 45.0% girls) aged 3–8 years (mean age: 5 years)	CEBQ	Children with more emotional temperaments showed more food- avoidant eating behaviors (*p* < 0.001). Elevated child BMI was related to additional food approach eating behaviors; however, BMI was not associated with child temperament (*p* < 0.001).	Haycraft et al., 2011 [49]
Cross-sectional study	1730 Canadian children (884 boys and 846 girls) aged 4–5 years	CEBQ	Substantial differentiations were noted between body weight status groups concerning food responsiveness, emotional overeating, enjoyment of foods, satiety responsiveness, slowness in eating, and food fussiness (*p* < 0.01 for all).	Spence et al., 2011 [50]
Cross-sectional study	4987 children (50.1% boys and 49.9% girls) aged 4 years from Netherlands	CEBQ	Elevated children’s food responsiveness, enjoyment of foods and parents’ constraints were related to a greater BMI (*p* < 0.001 for all). Emotional undereating, satiety responsiveness and fussiness of children as well as parents’ pressure to eat were inversely associated with child BMI (*p* < 0.001 for all).	Jansen et al., 2012 [51]
Cross-sectional study	3137 children (50.3% boys, 49.7% girls) aged 3 to 4 years from Netherlands	CEBQ	Children having enhanced levels of emotional difficulties exhibited decreased BMI-SDS after adjusting for relevant covariates for parent reports of emotional problems (*p* < 0.001).	Mackenbach et al., 2012 [52]
Longitudinal study	USA children aged 2 years with a one-year follow-up (323 children at baseline and 222 children one year later; gender not reported)	CEBQ	Mothers’ feeding methods exerted a crucial impact in the development of body weight increase and obesogenic eating behavior in children (*p* = 0.005, and *p* = 0.021, respectively)	Rodgers et al., 2013 [53]
Longitudinal study	521 children (255 boys and 266 girls) from Germany with a mean age of 8.26 years	EPIC	Substantial relationships of eating patterns with BMI were noted. Overweight children consciously restrain their eating (*p* < 0.001, and *p* < 0.0001, respectively).	Hirsch et al., 2014 [54]
Cross-sectional study	335 (48.7% boys and 51.3% girls) Brazilian children aged 6–10 years (mean age: 87.9 months)	CEBQ	Children presenting excessive body weight exhibited elevated scores at the CEBQ subscales related to “food approach”(*p* < 0.001) and elevated scores on two “food avoidance” subscales (*p* < 0.001, and *p* = 0.003) compared to normal-weight children.	dos Passos et al., 2015 [55]
Longitudinal study	995 Norwegian children aged 4 years, 760 children aged 6 years old, and 687 children aged 8 years (gender proportion not reported)	CEBQ	Children whose eating was remarkably induced by the sight and smell of foods prospectively showed elevated body weight increase (*p* < 0.001). Excessive body weight also predicted increased food approach behavior (*p* < 0.001).	Steinsbekk et al., 2015 [56]
Cross-sectional study	1058 Chilean children (49.2% boys and 50.8% girls) aged 7–10 years	CEBQ	A considerable association of eating behavior levels with BMI z-scores in children was noted (*p* < 0.0001). Children BMI was directly related to pro-intake eating behavior levels and inversely related to anti-intake eating behavior levels (*p* < 0.0001 for both).	Sánchez et al., 2016 [57]
Cross-sectional study	5426 children (46.0% boys and 54.0% girls) aged 9–11 years from 12 countries	EIES	Emotional eating was positively and consistently (across 12 study sites) related to a non-healthy dietary pattern (*p* < 0.0001). Emotional eating was not correlated with BMI (*p* = 0.493).	Jalo et al., 2019 [58]
Validity study	147 USA overweight or obese children (34.0% boys and 66.0% girls) aged 8–12 years (mean age: 10.4 years)	EES-C	The initial interpretative importance of the EES-C between treatment-seeking children affected by overweight or obesity should be assigned on a single general concept, and not on the 3 or 5 subconstructs (*p* < 0.05).	Kang Sim et al., 2019 [59]
Cross-sectional study	400 children aged 11–13 years from India (gender was not reported)	EES-C	EES levels were shown to be considerably elevated amongst caries-free subjects compared with those who exhibited mean “decayed and filled teeth”/“decayed missing and filled teeth (dft/DMFT)” score > 0 (*p* = 0.015, *p* = 0.001, and *p* = 0.076).	Goel et al., 2022 [60]

* PCSC: Perceived Competence Scales for Children, EIES: Emotion-Induced Eating Scale, DEBQ-C: Dutch Eating Behavior Questionnaire for Children, CEBQ: Child Eating Behavior Questionnaire, EPIC: Eating Patten Inventory for Children, MANOVA: Multivariate analysis of variance, TFEQ-R18: Three-Factor Eating Questionnaire-Revised 18, EES-C: Eating Scale—Adapted for Children and Adolescents.

**Table 3:** Clinical studies exploring the associations between mental disorders and children’s emotional eating.

Study Type	Study Population	Children EE Assessment *	Basic Results	References
Cross-sectional study	356 Latino (42.6%), African (21.6%) and Asian (19.2%) American children (43.0% boys and 57.0% girls) aged 8–12 years	DEBQ-C	There were considerable relationships between sleep onset latency and emotional eating (*p* = 0.030), depressive symptomology (*p* < 0.0001) and trait anxiety (*p* < 0.0001).	Nguyen-Rodriguez et al., 2010 [61]
Cross-sectional study	437 Belgium children (49.9% boys and 50.1% girls) aged 5–12 years (median age about 9.0 years)	DEBQ-C	Stressful events, negative emotions and problems were directly related to emotional eating and an unhealthier dietary pattern (*p* < 0.01).	Michels et al., 2012 [62]
Cross-sectional study	476 Swiss children (251 boys and 225 girls) aged 2–6 years (mean age: 3.89 years)	CEBQ	Children presenting difficulties in their temperament may have increased risk of emotional eating and body weight modulation troubles in later childhood (*p* < 0.001 for all).	Messerli-Bürgy et al., 2018 [63]
Cross-sectional study	241 overweight or obese children (90 boys and 151 girls) aged 7–11 years from USA	CBC	Child eating disorder pathology, parents’ use of psychological control, and reduced children personal social condition were substantially related to increased children’s depressive symptoms (*p* < 0.001 for all). Children eating disorders’ pathology and parents’ psychological management were significantly related to elevated child anxiety symptomatology (*p* < 0.001 for all).	Sheinbein et al., 2019 [64]
Cross-sectional study	1120 children (57.5% boys and 42.5% girls) aged 11–12 years from Taiwan	DEBQ-C	Those children who self-reported elevated anxiety and depressive levels exhibited increased stress events and emotional eating compared with those with reduced anxiety and depressive levels (*p* < 0.001, and *p* < 0.01, respectively).	Wu et al., 2020 [65]
Cross-sectional study	361 children (6.0% boys and 94.0% girls) aged 3–5 years from USA	CEBQ	Child food insecurity was only related to greater food responsiveness amongst children of parents stating elevated scores of perceived stress and decreased scores of family functionality (*p* = 0.04, and *p* = 0.01, respectively).	Eagleton et al., 2022 [66]
Longitudinal study	1136 children (53.3% boys and 46.7% girls) aged 2.5 years from USA with a follow-up at 8 years	DEBQ-C	Mechanical/distress feeding difficulties in children aged 2.5 years. No food refusal difficulties were related to ADHD, problematic behavior (OD/CD), and anxiety/depressive symptomatology at the age of eight years (*p* = 0.012, and *p* = 0.002, respectively).	Putnick et al., 2022 [67]
Cross-sectional study	349 Turkish children (128 boys and 221 girls) aged 9–11 years	EES-C	A positive, weak relationship was also observed between children’ age and emotional eating, anxiety–anger–disappointment subscales and perceived stress scores (*p* = 0.003, *p* = 0.001, and *p* < 0.001, respectively)	Doğan Güney et al., 2024 [68]

* DEBQ-C: Dutch Eating Behavior Questionnaire for Children, CEBQ: Child Eating Behavior Questionnaire, CBC: Child Behavior Checklist, EES-C: Eating Scale-Adapted for Children and Adolescents, ADHD: attention-deficit/hyperactivity, OD: Oppositional-defiant, CD: Conduct disorder.

**Table 4:** Clinical studies evaluating the association of children dietary habits with emotional eating.

Study Type	Study Population	Children EE Assessment *	Basic Results	References
Longitudinal study	400 USA twin children (44.3% boys and 55.5% girls) aged 4 years at the baseline and 322 children (38.5% boys and 61.5% girls) with a follow up at the age of 10 years	CEBQ	Satiety responsiveness, eating slowness, food fussiness, and emotional undereating were reduced, while food responsiveness, food enjoyment and emotional overeating were enhanced (*p* < 0.0001 for all).	Ashcroft et al., 2008 [69]
Case–control study	25 UK children (7 boys and 5 girls in the experimental group, 6 boys and 7 girls in the control group) aged 3–5 years	CFPQ	Children of mothers using foods for emotional modulation more often consumed sweet palatable foods in the absence of hunger than children of mothers using this feeding method rarely (*p* = 0.0008).	Blissett et al., 2010 [70]
Longitudinal study	782 second graders (50.7% boys and 49.3% girls) were followed up through third grade from USA	DEBQ-C	Children’s emotional modulation was substantially associated with both external and emotional eating within grades (*p* < 0.0001). Reactivity was more consistently associated with eating regulation than was inhibition (*p* < 0.0001).	Harrist et al., 2013 [71]
Case–control study	62 UK children (33 boys and 29 girls) aged 34–59 months (mean age 46.0 months)	CEBQ	Children presenting elevated levels of emotional undereating on the CEBQ consume less energy from crisps/potato chips and cookies (*p* < 0.05) throughout a negative mood state, but not during a neutral mood.	Blissett et al., 2019 [72]
Longitudinal study	Norwegian children followed up from age 4 (997 children) to age 6 (795 children), 8 (699 children) and 10 (702 children) years (gender not reported)	CEBQ	Temperament was implicated in the etiology of children’s eating patterns. Harmful affectivity influenced both ‘foods approach’ and ‘foods avoidant’ behavior (*p* < 0.001 for both)	Steinsbekk et al., 2020 [73]
Cross-sectional study	178 Italian children (54.5% boys and 45.5% girls) aged 8–9 years	CEBQ	Emotional overeating was positively related to both emotional symptoms and hyperactivity and inversely associated with peer problems (*p* < 0.01, *p* < 0.01, and *p* = 0.03, respectively). Emotional undereating was also directly related to the number of siblings and inversely correlated with high Mediterranean diet compliance (*p* = 0.04, and *p* = 0.02, respectively).	Buja et al., 2022 [74]
Cross-sectional study	408 Chinese children (52.2% boys and 47.8% girls) aged 6–36 months (mean age: 22.4 months)	CEBQ	Caregivers’ emotional and instrumental feeding was directly related to children’s intake of ultra-processed foods, a higher frequency of ultra-processed food consumption weekly, and a larger amount of ultra-processed food consumption weekly (*p* < 0.01 for all). Children’s elevated incidence of emotional undereating was related to their ultra-processed food consumption and a higher frequency of ultra-processed food consumption weekly (*p* < 0.01 for all).	An et al., 2022 [75]

* CFPQ: Comprehensive Feeding Practices Questionnaire, DEBQ-C: Dutch Eating Behavior Questionnaire for Children, CEBQ: Child Eating Behavior Questionnaire.

**Table 5:** Clinical studies assessing the impact of other diverse factors on children’s emotional eating.

Study Type	Study Population	Children EE Assessment *	Basic Results	References
Cross-sectional study	221 Chilean obese children with a mean age of 9.7 years (gender not reported)	CEBQ	The dimensions “slow eating”, “emotional eating”, “foods enjoyment” and “uncontrolling eating” were considerably related to some polymorphisms of leptin and leptin receptor (*p* < 0.01 for all).	Valladeres et al., 2014 [76]
Longitudinal study	Belgium Children (48.7% boys and 51.3% girls) were between 5 and 10 years old at baseline (308 children) and between 7 and 12 years old at follow-up (174 children)	DEBQ-C	Stress marker (overall cortisol output) was substantially related to elevated leptin concentrations, but only in girls and cross-sectionally (*p* = 0.029). Leptin was not identified as a considerable predictor of non-healthy food intake (*p* > 0.05).	Michels et al., 2017 [77]
Longitudinal study	2054 UK twin children aged 2 years which were followed up to 5 years (boys:girls ratio about 1:1)	CEBQ	Emotional overeating and emotional undereating were positively interrelated, and this relationship was clarified by common shared environmental influences (*p* < 0.001).	Herle et al., 2017 [78]
Longitudinal study	2402 UK twin children aged 2 years which were followed up to 5 years (boys:girls ratio about 1:1)	CEBQ	Genetic influences on emotional overeating were minimal compared to shared environmental influences (*p* < 0.001).	Herle et al., 2018a [79]
Longitudinal study	394 UK twin children aged 4 years (44.9% boys and 55.1% girls)	CEBQ	Genetic impact was not considerable, whereas shared environmental circumstances were implicated in the 71% variance in emotional overeating and 77% in emotional undereating (*p* < 0.01).	Herle al., 2018b [80]
Cross-sectional study	147 children (49.7% boys and 50.3% girls) aged 4–6 years from USA	SEE	The interconnection of 5-HTTLPR with impulsivity and negative affectivity considerably predicted body fat percentage. The interconnection of 5-HTTLPR with impulsivity and negative affectivity considerably predicted both total calorie intake and rate of overall calorie intake (*p* < 0.01).	Ohrt et al., 2020 [81]

* CEBQ: Child Eating Behavior Questionnaire, DEBQ-C: Dutch Eating Behavior Questionnaire for Children, 5-HTTLPR: Human serotonin transporter gene, SEE: Stress-induced emotional eating.

## Data Availability

Data are available upon request to the corresponding author.
